# Efficacy of Omaha system-based nursing management on nutritional status in patients undergoing peritoneal dialysis: A randomized controlled trial protocol

**DOI:** 10.1097/MD.0000000000023572

**Published:** 2020-12-18

**Authors:** Liqing Peng, Yuanyuan Gao, Rong Lu, Ruixiang Zhou

**Affiliations:** Department of Critical Care Medicine, Wuhan No.1 Hospital, Hubei, China.

**Keywords:** chronic kidney disease, peritoneal dialysis, Omaha system, protocol

## Abstract

The chronic kidney disease (CKD) patients may have a variety of complications during receiving peritoneal dialysis (PD). The malnutrition in CKD patients is related to their lower life quality, higher hospitalization rates, and higher risk of cardiovascular disease, as well as the increased morbidity and mortality. Hence, it is very important to monitor and then manage the nutritional status of CKD patients. Thus, we perform this randomized controlled study protocol to introduce a continuing nursing program based on Omaha system (OS) for the patients with CKD receiving PD treatment.

The randomized trial will be implemented from November 2020 to May 2021 and was granted through the Research Ethics Committee of Wuhan No.1 Hospital (2020003281). Two hundred patients meet inclusion criteria and exclusion criteria are included.

Patients who meet the following criteria will be selected: voluntary participation, aged 20 to 60; undergoing the regular PD treatment for at least 3 months. Patients will be excluded if the patients are in unstable status, or experience the intermittent PD or some other kinds of dialysis mode, have severe cachexia, infection, or malnutrition, or if they have mental disorders. In control group, patients are given routine treatment, containing general guidance associated with PD and the outpatient telephone calls from the clinical nurses during follow-up. In study group, the patients are given the continuous nursing treatment scheme based on OS. The clinical results are the biochemical parameters after intervention, anthropometry, as well as the subjective global assessment.

Table 1 reveals the clinical endpoints between the 2 groups.

This protocol can guide nurses to develop a nursing program based on evidence for patients with CKD receiving PD.

**Trial registration:**

This study protocol was registered in Research Registry (researchregistry6202).

## 1 Introduction

Chronic kidney disease (CKD) is defined through the National Institute of Diabetes and Digestive and Kidney Diseases.^[1,2]^ It refers to the continuous decline of renal function with the rate of glomerular filtration below 60 mL/min.^[3]^ This disease affects 8% to 16% of the world's population and it is generally ignored by clinicians and patients. CKD is more common in the low-income and middle-income countries than in the high-income countries.^[4]^ Globally, the most familiar causes of CKD are hypertension and diabetes, but there are some other causes, for instance, infections, glomerulonephritis, and the environmental exposures (e.g., pesticides, herbal medicines, and air pollution).^[5–7]^ Since 2007, the global CKD prevalence has increased by 34.2%. In 2017, CKD led to 123 million deaths.^[8]^

Peritoneal dialysis (PD) is a kind of effective treatment of renal replacement for the CKD patients.^[9,10]^ The outcomes of PD treatment leads to the removal of excess waste and fluid from the blood into dialysate and offers better health results in terms of life quality and survival rate in CKD patients. Due to the advantages of simple operation and high cost-effectiveness, PD is extensively utilized in many countries. At present, >270,000 patients worldwide are treated with PD, accounting for approximately 10% of all the dialysis patients.^[11]^ In addition, the annual percentage of patients undergoing PD worldwide has enhanced by 8%, higher than the percentage of hemodialysis.^[12]^

The CKD patients may have a variety of complications, for instance, catheter dysfunction, infection, malnutrition, and fatigue. The malnutrition in CKD patients is related to their lower life quality, higher hospitalization rates, and higher risk of cardiovascular disease, as well as the increased morbidity and mortality. Hence, it is very important to monitor and then manage the nutritional status of CKD patients receiving PD. Thus, we perform this randomized controlled study protocol to introduce a continuing nursing program based on Omaha system (OS) for the patients with CKD receiving PD treatment.

## 2 Methods

The experiment will be implemented from November 2020 to May 2021 at Wuhan No.1 Hospital. The experiment was granted through the Research Ethics Committee of Wuhan No.1 Hospital (2020003281) and recorded in research registry (researchregistry6202). Prior to registration, the patients who are recruited receive written informed consent.

### 2.1 Inclusion criteria and exclusion criteria

Patients who meet the following criteria will be selected: voluntary participation, aged 20 to 60; undergoing the regular PD treatment for at least 3 months. Patients will be excluded if the patients are in unstable status, or experience the intermittent PD or some other kinds of dialysis mode, have severe cachexia, infection or malnutrition, or if they have mental disorders.

### 2.2 Randomization

Two patients meet inclusion criteria and exclusion criteria are included. In the random envelope, a random number is assigned to whole patients through the random-number table, and the distribution result is invisible. Patients are assigned randomly to conservative group (n = 100) and surgical group (n = 100).

### 2.3 Intervention

In control group, patients are given routine treatment, containing general guidance associated with PD and the outpatient telephone calls from the clinical nurses during follow-up. In study group, the patients are given the continuous nursing treatment scheme based on OS as shown below. Education: the self-management health lecture of PD are held, mainly including the knowledge of environment preparation and PD operation, involving the process of PD operation, the dialysate heating approach, aseptic operation technology, the times of perfusion and dialysis drainage, the complications related to PD, and the PD tube skin care. In the aspect of dietary management information, low-phosphorus diet, low-salt diet, high-quality protein diet, the food kinds and importance of the intake of potassium are the major contents. After finishing the lecture, a group discussion is organized to in-depth investigate the health problems raised by the patients. The materials of health education are publicized in Wechat group twice a week. And the materials focus on nutrition management and PD. Moreover, the video for the process of PD operation is made and played in Wechat group to help the patients understand the operation process. Procedure and treatments: firstly, the self-designed evaluation table based on Omaha Question Classification System is utilized to identify the nursing problems of patients, involving cognitive, nutrition, emotional, and the economic problems. Afterwards, the objectives of nursing intervention are established and the intervention strategies are conducted (for instance, personalized health education, guidance, or consultation). Case management: the patients are instructed on how to record their own diet in their self-management manual, involving the quantities and types of daily diet. According to the dietary records, clinical nutritionists make dietary suggestions, and then formulate the target diet. Surveillance: in the process of Wechat, outpatient, and telephone follow-up, the weight, the management of drug, patient's diet, sleep, working conditions as well as some other factors are assessed to determine the unsatisfied nursing needs of patients and carry out targeted intervention.

### 2.4 Clinical endpoints

The clinical results are the biochemical parameters, anthropometry, as well as the subjective global assessment after intervention.

### 2.5 Statistical analysis

All the data can be recorded into the software of Microsoft Excel 2010, and the data are analyzed through utilizing the software of IBM SPSS Statistics for Windows, version 20 (IBM Corp., Armonk, NY). Subsequently, all data are represented via the proper characteristics, for instance, median, mean, and percentage. The categorical variables and continuous variables are respectively analyzed using independent *t* tests and chi-squared-tests. *P* value <.05 indicates that there is statistical significance.

## 3 Results

Table 1 indicates the clinical outcomes between 2 groups.

**Table 1:** The clinical endpoints between the two groups.

Variables	Study group (n = 100)	Control group (n = 100)	*P* value
Well nourished			
Moderately malnourished			
Severely malnourished			
Body mass index			
Triceps skin-fold thickness			
Mid-arm muscle circumference			
Handgrip strength			
Hemoglobin			
Albumin			
Total cholesterol			

## 4 Discussion

The technological innovations of PD have greatly decreased the complications related to treatment, so that patients can maintain PD for a long time.^[13,14]^ Nevertheless, patients with PD are characterized through the high risk of malnutrition, with the estimated prevalence of 40%.^[15,16]^ The former studies have suggested continuous follow-up plans and health care in CKD patients treated for PD, but have failed to focus on the management of nutrition.^[17]^ There is a lack of the tailored nutritional interventions for the patients and adequate monitoring by medical practitioners. This may lead to self-management of nutritional intake by patients and can cause confusion as they adjust to novel eating habits. In early 1970s, the OS is established, which contains 3 fundamental steps: problem, intervention, and result.^[18]^ Few researches have used the OS to deal with nutritional problems in patients with CKD receiving PD. Hence, its influences on improving the nutritional status of patients need to be in-depth investigated in clinical practice.

## 5 Conclusion

This protocol can guide nurses to develop a nursing program based on evidence for patients with CKD receiving PD.

## Author contributions

**Data curation:** Yuanyuan Gao.

**Formal analysis:** Yuanyuan Gao.

**Funding acquisition:** Ruixiang Zhou.

**Methodology:** Rong Lu.

**Software:** Rong Lu.

**Writing – original draft:** Liqing Peng.
